# Expression of lysophosphatidic acid receptors in the human lower esophageal sphincter

**DOI:** 10.3892/etm.2013.1439

**Published:** 2013-12-06

**Authors:** YONG FENG, JUN-FENG LIU

**Affiliations:** Department of Thoracic Surgery, Fourth Hospital of Hebei Medical University, Shijiazhuang, Hebei 050011, P.R. China

**Keywords:** clasp fibers, human, lower esophageal sphincter, lysophosphatidic acid receptors, sling fibers

## Abstract

Lysophosphatidic acid (LPA) is a bioactive lipid that is involved in a variety of physiological and pathological processes occurring in the gastrointestinal tract. It acts via six distinct types of receptors, LPA1, LPA2, LPA3, LPA4, LPA5 and LPA6, which belong to the family of G protein-coupled receptors. The aim of the present study was to detect the expression of the LPA receptors in the human lower esophageal sphincter (LES). Quantitative polymerase chain reaction and western blotting were used to analyze the expression of LPA1-6 receptors in sling and clasp fibers from the human LES. The results showed that the protein and mRNA expression levels of various LPA receptors were significantly different. Specifically, the mRNA and protein expression levels of the LPA1 receptor were higher compared with those of the other receptors. The prevalence of the LPA1 receptor mRNA and protein indicates that the LPA1 receptor is likely to be involved in the regulation of human LES functions.

## Introduction

Lysophosphatidic acid (LPA) is a bioactive lipid mediator that is released by activated platelets and is constitutively present in serum (1,2). It has been identified to be a potent phospholipid messenger with a variety of biological actions, which include cell proliferation, survival and migration, wound healing, platelet aggregation, vascular remodeling, neurite retraction, differentiation inhibition/reversal, membrane depolarization, formation of focal adhesion and stress fibers (3–8), blood pressure regulation (9) and smooth muscle contraction (10–13).

LPA exhibits its functions mainly through binding to its specific G protein-coupled receptors (GPCRs). Currently, there are six GPCRs identified as specific receptors for LPA that are referred to as LPA1-6 (14–20). LPA1-3 were identified as members of the endothelial differentiation gene (Edg) subfamily of GPCRS, as they share a high homology with each other (21). By contrast, LPA4-6 belong to the non-Edg family (22–24).

It has been demonstrated through studies in guinea pigs, rats and cats that LPA receptors are identifiable throughout the length of the mammalian gastrointestinal tract (11,12,25). However, there is less information available concerning the distribution of LPA receptors in the lower esophageal sphincter (LES) and the studies have been mainly confined to animals.

Esophageal motility disorders, including achalasia and diffuse esophageal spasm, are characterized by the incomplete passage of swallowed contents into the stomach. Such conditions are often accompanied by incomplete or failure of LES relaxation during swallowing. Specific treatments for such conditions are not known at present.

The aim of the present study was to examine the expression of the LPA receptors in the human LES, in particular within the clasp and sling fibers of the LES complex. In addition, the potential implications of the results obtained for the respective roles of the LPA receptors in the physiological regulation of human LES function were considered.

## Materials and methods

### Patients and tissue retrieval

The experimental protocol was approved by the Research Ethics Committee of the Fourth Hospital (Hebei Medical University, Shijiazhuang, China). The muscle strips were collected from 15 patients who underwent an esophagectomy for mid-third esophageal carcinoma in the Department of Thoracic Surgery (Fourth Hospital) between January 2012 and July 2012. There were 9 males and 6 females, with an average age of 64 years (range, 55–68 years). Patients with a history of gastroesophageal reflux disease or esophageal motor disorders were excluded from the study. Each specimen was resected *en bloc* in the operating room and placed immediately in ice-cold Krebs solution, the composition of which has been described previously (26). Specimens were not included in this study if the segment contained a macroscopically visible tumor.

In the laboratory, fresh esophagogastric junction specimens collected in the operating room were immediately placed in 4°C Tris-buffered saline (TBS). Following washing with 37°C Krebs solution, specimens were pinned on a wax plate containing TBS, with a continuous mixed gas of 95% O_2_ and 5% CO_2_. The mucosa and submucosa were then gently removed by sharp dissection. The sling fibers, clasp fibers and circular muscle strips of the esophagus and stomach were separated and prepared into 2–4 × 8–12-mm muscle strips. The LES was recognized as a thickened band of circular muscle at the gastroesophageal junction.

The gastric sling and clasp fibers were identified as thickened bands of circular oriented smooth muscle in the gastric cardia, adjacent to the greater and lesser curvature of the stomach, respectively. The sling and clasp muscle strips were prepared using a method described previously (27,28). Circular muscle strips from the esophagus and stomach were prepared as controls. These circular muscle strips were obtained 3 cm proximal and distal to the gastroesophageal junction. The circular muscle was not excised to the full depth of this layer to avoid including the myenteric plexus and the longitudinal muscle in the wall of the esophagus and stomach. The dissected muscle strips were frozen in liquid nitrogen and stored at −80°C for subsequent RNA extraction. Written informed consent was obtained from the patients.

### RNA isolation and reverse transcription-polymerase chain reaction (RT-PCR) for LPA receptors

Tissue was homogenized in TRIzol reagent at a ratio of 100 mg tissue to 1 ml TRIzol (Invitrogen Life Technologies, Carlsbad, CA, USA) and then centrifuged at 12,000 × g for 5 min. Total RNA was extracted by acid guanidinium thiocyanate-phenol-chloroform extraction. The quality of the RNA was verified by agarose gel electrophoresis using ethidium bromide staining. First-strand cDNA synthesis (reaction volume, 20 μl; RevertAid First Strand cDNA Synthesis kit; Thermo Scientific, Waltham, MA, USA), using 2 mg RNA, was performed in the presence of RevertAid Moloney Murine Leukemia Virus (M-MuLV; Thermo Scientific). Reverse transcription (Fermentas, Glen Burnie, MD, USA) was performed using 0.5 mg oligo(dT)_18_ and diethylpyrocarbonate (DEPC)-treated water to reach a volume of 11 μl. Samples were then incubated at 70°C for 5 min, prior to being chilled on ice. Next, 4 μl reaction buffer (5X), 2 μl 4 dNTP mix (10 mM) and 20 units RNasin were added, using DEPC-treated water to reach a 19 μl volume and then incubated at 37°C for 5 min. Finally, 200 units (1 μl) of RevertAid M-MuLV reverse transcriptase was added and the reaction mixture was incubated at 42°C for 60 min. Following this, the reaction was terminated and held at 70°C for 10 min.

PCR amplification of the cDNA was performed using primers designed specifically to match the mRNA of the LPA receptors (primers listed in Table I). A volume of 2 μl cDNA reaction mixture was used in each PCR, which was performed in a 20 μl reaction volume. The amplification conditions for each LPA receptor were different. PCR conditions: LPA1, 36 cycles of 94°C for 5 min, 94°C for 30 sec, 58°C for 40 sec and 72°C for 1 min, followed by 72°C for 5 min; LPA2, 38 cycles of 94°C for 5 min, 94°C for 30 sec, 56°C for 40 sec and 72°C for 1 min, followed by 72°C for 5 min; LPA3, 40 cycles of 94°C for 5 min, 94°C for 30 sec, 57°C for 40 sec and 72°C for 1 min, followed by 72°C for 5 min; LPA4, 36 cycles of 94°C for 5 min, 94°C for 30 sec, 57°C for 40 sec and 72°C for 1 min, followed by 72°C for 5 min; LPA5, 42 cycles of 94°C for 5 min, 94°C for 30 sec, 60°C for 40 sec and 72°C for 1 min, followed by 72°C for 5 min; LPA6, 38 cycles of 94°C for 5 min, 94°C for 30 sec, 58°C for 40 sec and 72°C for 1 min, followed by 72°C for 5 min; β-actin, 23 cycles of 94°C for 5 min, 94°C for 30 sec, 58°C for 40 sec and 72°C for 1 min, followed by 72°C for 5 min. A negative control in which all the components of the reaction were added, with the exception of the cDNA template, was tested in parallel with each sample to identify any risk of false positive results. Amplified products were electrophoresed on a 1.5% agarose gel and photographed under a UV transilluminator. PCR, including positive and negative controls, was performed in triplicate with cDNA extracted from fifteen independent specimens.

### Quantitative PCR (qPCR) for LPA receptors

Primers were designed specifically to match the mRNA of the LPA receptors (primers listed in Table II). Analysis of the LPA receptor mRNA expression was carried out by qPCR using TransStart™ Top Green qPCR SuperMix (Beijing TransGen Biotech Co., Ltd, Beijing, China) and an ABI 7500 PCR system (Applied Biosystems, Inc., Foster City, CA, USA). The final reaction mixture of 25 μl consisted of 1 μl diluted cDNA, 12.5 μl 2X TransStart Top Green qPCR SuperMix, 0.5 μl Passive Reference Dye, 10 μl ddH_2_O, 0.5 μl forward primer and 0.5 μl reverse primer. The reactions were performed in triplicate according to the manufacturer’s instructions. All the reactions were performed in 96-well plates in duplicate. ABI 7500 conditions were designed as follows: Initial denaturation at 94°C for 30 sec, followed by 42 cycles of 94°C for 5 sec, 60°C for 34 sec and melt curve stage. The relative expression levels of each mRNA were determined using the ABI 7500 software (version 2.0.5). The precise amount of total cDNA added to each reaction mix and its quality are difficult to assess; therefore, the expression level of the gene of interest in a given specimen was computed relative to the level of β-actin mRNA, used as an invariant standard control to normalize the variations between the sample preparations. The threshold cycle (Ct) was used for quantification of the input target numbers. The normalized expression levels of the target gene were calculated by the 2^−Δ(ΔCt)^ method, where ΔCt = Ct_target gene_ − Ct_β-actin_ and Δ(ΔCt) = ΔCt_test sample_ − ΔCt_control_.

### Western blot analysis of LPA receptors

Total proteins were extracted from the muscle strips using a protein extraction kit (Solarbio, Beijing, China). Protein concentration was determined with a colorimetric BCA protein assay reagent (Multisciences, Hangzhou, China). Following denaturation at 100°C for 10 min, aliquots of protein samples (30 μg) were separated by electrophoresis on SDS-polyacrylamide gel (10% separation gel and 4% pycnotic gel) at 150 V for 1 h and then transferred onto a polyvinylidene difluoride membrane. The membrane was blocked for 1 h with 5% non-fat milk in Tris-buffered saline with Tween 20 (TBST) at room temperature and incubated with a specific primary antibody [dilutions: rabbit anti-LPA1R, 1:1,000 (GeneTex, San Antonio, CA, USA); mouse anti-LPA2R, 1:2,000 (Abcam, Cambridge, UK); rabbit anti-LPA3R, 1:2,000 (GeneTex); rabbit anti-LPA4R, 1:2,000 (Abcam); rabbit anti-LPA5R, 1:2,000 (Abcam); rabbit anti-LPA6R, 1:2,000 (Abcam); and rabbit anti-β-actin, 1:10,000 (GeneTex)] at 4°C overnight. Next the membrane was washed three times with TBST at room temperature for 30 min in total and then incubated with a secondary antibody (1:2,000, anti-rabbit IgG; Rockland, Philadelphia, PA, USA) for 1 h. Following three washes with TBST, the membrane was analyzed using an infrared fluorescence imaging instrument (Odyssey, model: 9120; LI-COR, Lincoln, NE, USA). The relative expression level of each protein was normalized against the value of β-actin.

### Statistical Analysis

Data are expressed as mean ± SD. SPSS 19.0 (SPSS, Inc., Chicago, IL, USA) was used for statistical analysis. Differences in mRNA expression were analyzed with one-way ANOVA and the Student-Newman-Keuls multiple range test was used for comparisons within groups. P<0.05 was considered to indicate a statistically significant difference.

## Results

### Characterization of mRNA encoding six LPA receptors

**Figure 1:**
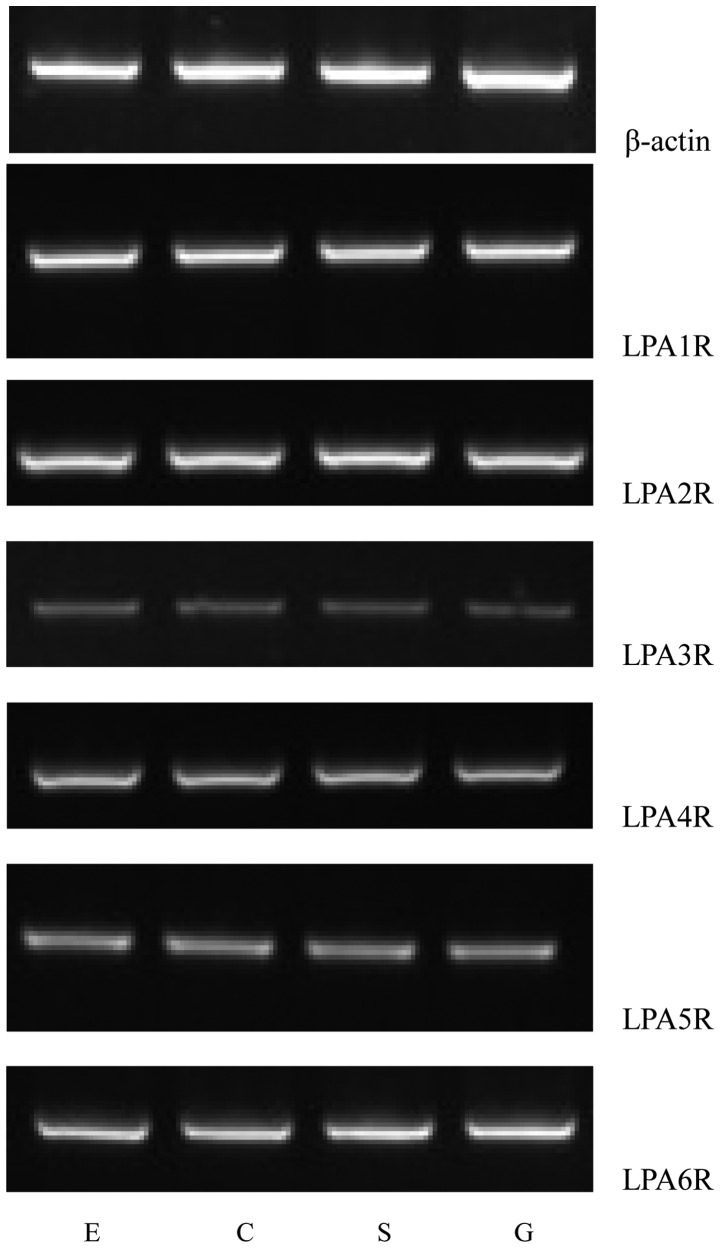
mRNA expression of LPA receptors in the sling and clasp fibers of the LES and circular muscle strips from the esophagus and stomach. A representative example of the RT-PCR products specific for each LPA receptor mRNA. LPA, lysophosphatidic acid; LES, lower esophageal sphincter; RT-PCR, reverse transcription-polymerase chain reaction; S, sling fibers; C, clasp fibers; E, circular muscle strips of esophagus; G, stomach.

Using RT-PCR, the mRNA expression levels of six LPA receptors in the human LES were observed, as illustrated in Fig. 1. Distinct bands of the expected sizes were detected for each of the six LPA receptor mRNAs and their levels of expression were apparently different. Similar results were obtained in all PCR assays performed on mRNA extracted from the fifteen patients. The primer pair designed to recognize the LPA1R mRNA generated a strong band, indicating high expression levels of the LPA1R mRNA in the human LES. The LPA6R mRNA also generated a comparatively strong band. However, the primer pairs for the LPA4R, LPA5R and LPA2R mRNA produced relatively weak bands. The lowest apparent mRNA expression level was observed for the LPA3 receptor (Fig. 1).

### Quantification of LPA receptor mRNA expression

**Figure 2:**
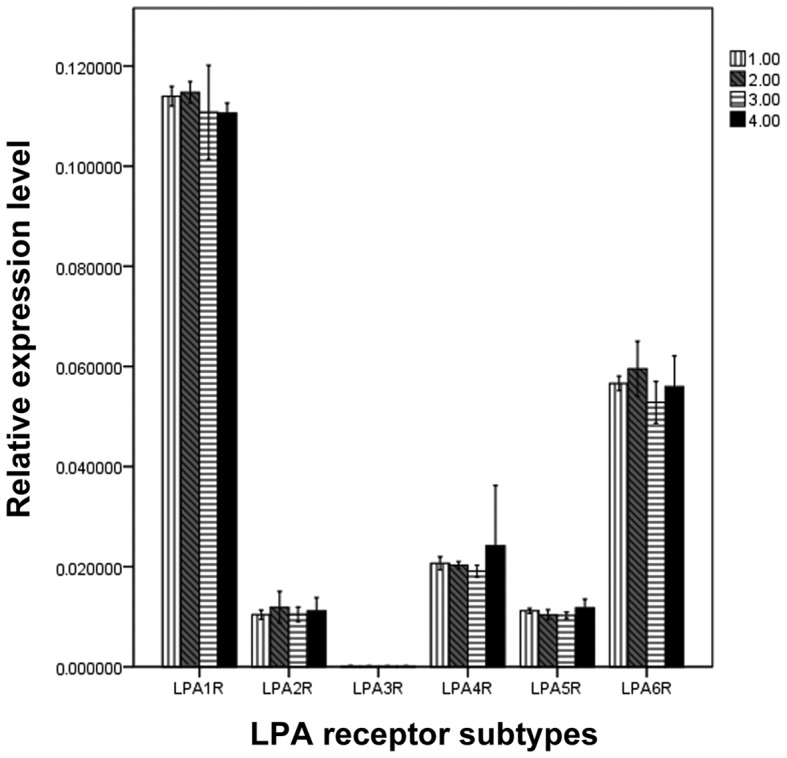
Quantitative determination of the mRNA expression levels of the various LPA receptors in the sling and clasp fibers of the LES and circular muscle strips from the esophagus and stomach. Bar chart showing the relative mRNA expression levels of the various LPA receptors normalized against β-actin mRNA expression in fifteen independent subjects. There were significant differences between the LPA receptor subtypes in the same muscle strip (P<0.05), but no significant differences for each subtype across various muscle strips (P>0.05). LPA, lysophosphatidic acid; LES, lower esophageal sphincter; 1.00, circular muscle strips of esophagus; 2.00, clasp; 3.00, sling; 4.00, circular muscle strips of stomach.

To compare the levels of different LPA receptor mRNA, qPCR was performed (Fig. 2). Significant differences were demonstrated when the mRNA expression levels of various LPA receptors were compared in the same muscle strips (F, 61.034; P=0.000). The rank order of the expression was as follows: LPA1R>LPA6R>LPA4R=LPA2R=LPA5R=LPA3R. However, no significant difference was identified in the mRNA expression levels of the LPA receptors between the four muscle strips. (F, 0.201; P=0.895; Fig. 2).

### Expression of LPA receptor proteins

**Figure 3:**
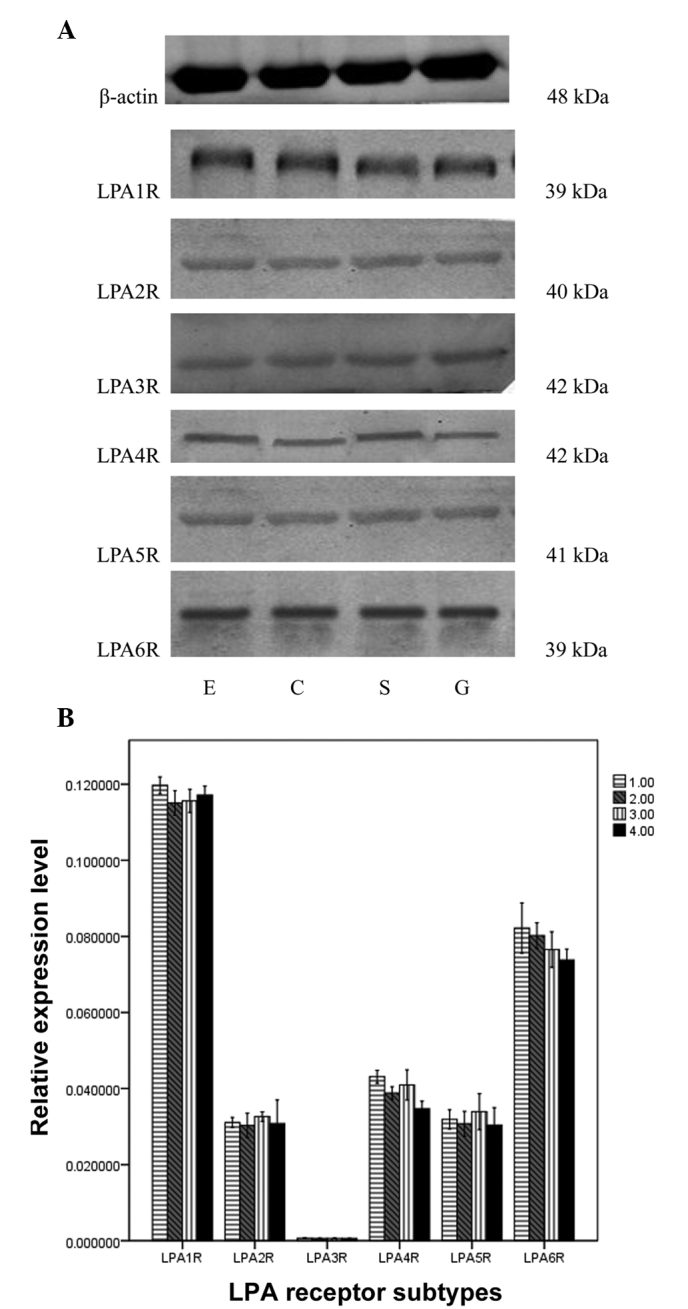
Expression of LPA receptor subtypes by western blotting in the sling and clasp fibers of the LES, circular muscle strips of esophagus and stomach. (A) Bands of the β-adrenoceptor subtype in S, C, E and G were identified with western blotting. (B) IOD values of the bands. There were significant differences between the β-adrenoceptor subtype in the same muscle strip (P<0.05), but no significant difference in a single subtype between the four muscle strips (P>0.05). LPA, lysophosphatidic acid; LES, lower esophageal sphincter; IOD, integrated optical density; S, sling fibers; C, clasp fibers; E, circular muscle strips of esophagus; G, stomach; 1.00, circular muscle strips of esophagus; 2.00, clasp; 3.00, sling; 4.00, circular muscle strips of stomach.

LPA1R, LPA2R, LPA4R, LPA5R and LPA6R protein expression was identified. Significant differences in the integrated optical density (IOD) values for the different LPA receptors in the same muscle strip were observed (F, 1,224.659; P=0.000). The rank order of the IOD values was as follows: LPA1R>LPA6R>LPA4R=LPA2R=LPA5R=LPA3R. No significant differences in IOD values were identified among the four muscle strips. (F, 0.039; P=0.990; Fig. 3).

## Discussion

LPA is a bioactive phospholipid with diverse biological functions in numerous tissues and cells. These functions are mediated by LPA receptors, which are significant members of the seven transmembrane domain GPCR family. LPA receptors are widely present in the mammalian central nervous system, including the brain, cardiovascular system and gastrointestinal tract and are significant physiological modulators.

Various esophageal motility disorders, including achalasia, diffuse esophageal spasm and nutcracker esophagus, are all associated with motor disorders of the LES. Previous studies have demonstrated that the regulatory mechanism of the LES involves various receptors, neurotransmitters and signal transduction pathways. CCK receptors, muscarinic receptors and dopamine receptors have demonstrated a role in the regulation of the LES (26–28).

The LPA receptors, widely distributed in the smooth muscles of the body, including gastrointestinal smooth muscle, are closely associated with the motility and secretion of the gastrointestinal tract. Pharmacological effects of LPA on various regions of the gut have been identified (11–13). Previous studies have also identified the expression of LPA receptors in the gastrointestinal tract (15,17–19,25,29). For instance, Lee *et al* demonstrated that the LPA1 receptor was significant in the regulation of the cat LES by pharmacological study (25). An *et al* identified the expression of the LPA2 receptor throughout the human gastrointestinal tract (15). LPA3 receptor was identified in the gastric smooth muscle by Sriwai *et al*(29) and Noguchi *et al*(17) identified the expression of the LPA4 receptor in the gut. Lee *et al*(19) and Kotarsky *et al*(18) showed that the LPA5 receptor was abundant in the gastrointestinal tract. These observations prompted the hypothesis that LPA receptors are located throughout the gastrointestinal tract. The present study was designed to determine whether LPA receptors exist in the human LES. The LES is a complex structure composed of clasp and sling fiber muscle strips in the gastric cardia and circular muscle fibers in the distal end of the esophagus, immediately above the gastroesophageal junction. In the present study, the clasp and sling fiber muscle strip component of the LES was specifically investigated.

Several studies carried out in various human tissues have indicated that the LPA3 receptor is not present in the gastrointestinal tract (16,30). However, the present study has clearly identified that the LPA3R is present in the esophageal body, human LES and stomach. The results obtained are in agreement with data obtained in previous studies performed in gastric smooth muscle (29). The differences in LPA receptor expression in the gastrointestinal tract, in the current study and previous studies, are not fully understood and require further study. In addition, in the present study, the LPA1 receptor was expressed at higher levels than the other receptors and the LPA2 receptor was also identified in the gastrointestinal tract. By contrast, a previous study has shown that the LPA1 and LPA2 receptors are not present in gastrointestinal smooth muscle (15). Differences between the current observations and those of other studies may be due to species differences. In the previous studies, LPA receptors were evaluated in animals, including rats, cats and guinea pigs. The present study detected the distribution of LPA receptors in the human LES and is, to the best of our knowledge, the first study to identify LPA receptor mRNA expression in the human LES.

Among the LPA receptors, the LPA1 receptor was identified to be the most highly expressed in the LES in the present study. The study suggests a major involvement of LPA1 receptor in the regulation of human gastrointestinal functions. It was also observed that the expression of the six LPA receptors between the four muscle strips did not differ.

The use of LPA receptor agonists and antagonists has shown that the LPA1 receptor blocks the relaxation of LES (25). Moreover, several studies show that LPA stimulates the contraction of smooth muscles (10–13). On the basis of previous pharmacological evidence, the expression of LPA receptors in the human LES, as demonstrated in the present study, implies an integral role in the modulation of the motility balance of the LES.

The present study is, to the best of our knowledge, the first to identify LPA receptor mRNA and protein expression in the human LES. Although little information on the physiological and pharmacological effects of the LPA receptors on the LES is available, the detection of LPA receptors in this study supports the concept that the LPA receptors are significant modulators of esophageal motility. Information concerning the functional role of the LPA receptors remains fragmented and requires further investigation. Future development of more specific ligands, as well as the use of gene deletion animal models, including knockout mice for each LPA receptor, are likely to allow precise evaluation of the physiological and pharmacological importance of the LPA receptors in the LES.

## Figures and Tables

**Table I:** Primers used for reverse transcription polymerase chain reaction.

Gene	Primer pair sequence (sense/antisense)	Product size (bp)
LPA1R	5′-ATC GGG ATA CCA TGA TGA GT C-3′ 5′-TCC GTT CTA AAC CAC AGA GTG-3′	342
LPA2R	5′-GTC CTC ATT ACC CAG TCA TAC CG-3′ 5′-CTG ATG GAC TCC ACC CTT TAG CT-3′	426
LPA3R	5′-TGT CAA CCG CTG GCT TCT-3′ 5′-CAG TCA TCA CCG TCT CAT TAG-3′	437
LPA4R	5′-GTGGCGGTATTTCAGCCTCT-3′ 5′-GAGTTGCAAGGCACAAGGTG-3′	402
LPA5R	5′-GATTCCGCCCTCTGAACACA-3′ 5′-AACCTGGTGCTCTTCAGCTC-3′	409
LPA6R	5′-TGGGTTGGACTCGTTGACTG-3′ 5′-TTTCGGACTTTGAGGACGCA-3′	458
β-actin	5′-TCC CTG GAG AAG AGC TAC GA-3′ 5′-ATC TGC TGG AAG GTG GAC AG-3′	362

LPAnR, lysophosphatidic acid receptor n.

**Table II:** Primers used for real-time quantitative polymerase chain reaction.

Gene	Primer pair sequence (sense/antisense)	Product size (bp)
LPA1R	5′-AAT CGG GAT ACC ATG ATG AGT CTT-3′ 5′-CCA AGG AGT CCA GCA GAT GAT AAA-3′	77
LPA2R	5′-CAG CCT GGT CAA GAC TGT TGT-3′ 5′-TGC AGG ACT CAC AGC CTA AA-3′	104
LPA3R	5′-ACG GTG ATG ACT GTC TTA GGG-3′ 5′-CAC CTT TTC ACA TGC TGC AC-3′	113
LPA4R	5′-AAA GAT CAT GTA CCC AAT CAC CTT-3′ 5′-CTT AAA CAG GGA CTC CAT TCT GAT-3′	139
LPA5R	5′-CGC CAT CTT CCA GAT GAAC-3′ 5′-TAG CGG TCC ACG TTG ATG-3′	66
LPA6R	5′-GGT AAG CGT TAA CAG CTC CCA CT-3′ 5′-TTT GAG GAC GCA GAT GAA AAT GT-3′	139
β-actin	5′-ATG AAG ATC AAG ATC ATT GCT CCTC-3′ 5′-ACA TCT GCT GGA AGG TGG ACA-3′	94

LPAnR, lysophosphatidic acid receptor n.
